# Biomarker evaluation for prognostic stratification of patients with COVID-19: the added value of quantitative chest CT

**DOI:** 10.2217/bmm-2021-0536

**Published:** 2022-02-18

**Authors:** Roberto M Ferreira, Gabriel SS de Oliveira, João RF da Rocha, Felipe de MC Ribeiro, João J Stern, Rangel de S Costa, Ricardo N Lemgruber, Júlia FP Ramalho, Alexandre CP de Almeida, Pedro PN Sampaio, João Mansur Filho, Ricardo AC Lima

**Affiliations:** ^1^Samaritano Hospital, Rua Bambina 98, Botafogo, Rio de Janeiro, RJ, 22251-050, Brazil; ^2^Federal University of Rio de Janeiro, Edson Saad Heart Institute, Rua Rodolpho Paulo Rocco 255, Ilha do Fundão, Rio de Janeiro, RJ, 21941-913, Brazil; ^3^Felippe Mattoso Clinic, Fleury Group, Rua Bambina 98, Rio de Janeiro, RJ, 22251-060, Brazil; ^4^Department of General Surgery, Federal University of The State of Rio de Janeiro, Rua Silva Ramos 32, Tijuca, Rio de Janeiro, RJ, 20270-330, Brazil

**Keywords:** biomarkers, coronavirus, CRP, D-dimer, prognosis, tomography, troponin

## Abstract

**Aim:** Pulmonary disease burden and biomarkers are possible predictors of outcomes in patients with COVID-19 and provide complementary information. In this study, the prognostic value of adding quantitative chest computed tomography (CT) to a multiple biomarker approach was evaluated among 148 hospitalized patients with confirmed COVID-19. **Materials & methods:** Patients admitted between March and July 2020 who were submitted to chest CT and biomarker measurement (troponin I, D-dimer and C-reactive protein) were retrospectively analyzed. Biomarker and tomographic data were compared and associated with death and intensive care unit admission. **Results:** The number of elevated biomarkers was significantly associated with greater opacification percentages, lower lung volumes and higher death and intensive care unit admission rates. Total lung volume <3.0 l provided further stratification for mortality when combined with biomarker evaluation. **Conclusion:** Adding automated CT data to a multiple biomarker approach may provide a simple strategy for enhancing risk stratification of patients with COVID-19.

Although most patients with COVID-19 have a benign clinical course, severe manifestations may occur in approximately 20% of cases [1]. Older age, hypertension, diabetes, obesity and underlying cardiovascular disease are associated with worse outcomes, though critical complications have also been reported in previously healthy adults [1]. The current pandemic has demanded that healthcare professionals become increasingly efficient in identifying high-risk clinical features in order to adequately allocate medical resources. Severe cases have been defined by the presence of dyspnea, tachypnea (respiratory rate ≥30 breaths per minute), blood oxygen saturation less than 93%, a ratio of the partial pressure of arterial oxygen to the fraction of inspired oxygen (Pao2:Fio2) of less than 300 mmHg or pulmonary infiltrates covering more than 50% of both lungs, in the first 48 h of symptom onset [2]. However, accurate risk stratification is particularly important in the emergency department even before these features develop.

Previous reports have suggested that the extent of pulmonary compromise may be predictive of mortality in patients with COVID-19. Chest computed tomography (CT) has consistently revealed greater opacification percentages and ground glass (GG) lesions in the presence of severe presentations [3–5]. In this context, automated quantification of CT disease burden may provide further assistance in standardizing the evaluation of pulmonary compromise and thus refining prognostic stratification [6,7].

While respiratory impairment represents a vital aspect of COVID-19, the systemic nature of the disease’s pathophysiology demands that other components which also affect clinical outcomes are routinely evaluated. As an example, venous thromboembolism is a frequent complication in hospitalized patients, and is associated with higher D-dimer levels, despite adequate prophylaxis [8]. However, in order to account for the variety of potential complications, a multiple biomarker approach utilizing high-sensitivity troponin I (hs-TnI), D-dimer and C-reactive protein (CRP) has also been proposed for predicting outcomes, both independently or utilizing a simple algorithm containing all three measurements [9–11]. Each of these markers represents an important feature of the disease’s pathophysiology, including myocardial injury, COVID-19-associated coagulopathy and systemic inflammation [10–13]. Still, the extent by which pulmonary disease burden and these three components interact and affect prognosis remains uncertain. The aim of this study was to evaluate the prognostic value of adding automated quantitative chest CT data to a multiple biomarker approach among hospitalized patients with COVID-19.

## Materials & methods

Consecutive patients with symptomatic COVID-19 admitted between 12 March and 8 July 2020 were retrospectively analyzed by medical chart review. Only those with a positive PCR result for SARS-CoV-2 were screened for inclusion. The study population was comprised by patients ≥18 years of age with recent onset (≤14 days) upper and/or lower respiratory symptoms, who were submitted to chest CT in addition to serum hs-TnI, D-dimer and CRP measurements on admission. Ongoing acute coronary syndromes during initial medical evaluation were the only exclusion criteria. Clinical and laboratory information were collected by four trained physicians who were blinded to chest CT results. A prespecified form with detailed instructions was elaborated for data extraction. The accuracy of data collection was confirmed by a fifth physician by random evaluation of completed forms from all reviewers.

Myocardial injury was defined by hs-TnI levels above the 99th percentile upper reference limit of the assay (>34 pg/ml), in accordance to previous reports indicating a prognostic value of this criterion [9]. Given the high prevalence of D-dimer and CRP elevation among hospitalized patients with COVID-19, both thresholds were defined according to the median values found for each biomarker among all included patients (D-dimer ≥810 ng/ml [reference <500 ng/ml] and CRP ≥7.4 mg/dl [reference <0.5 mg/dl]). This method has also demonstrated predictive value in COVID-19 [11]. Patients were subsequently divided into four groups according to the number of elevated biomarkers: 0, I, II or III.

**Figure 1.**
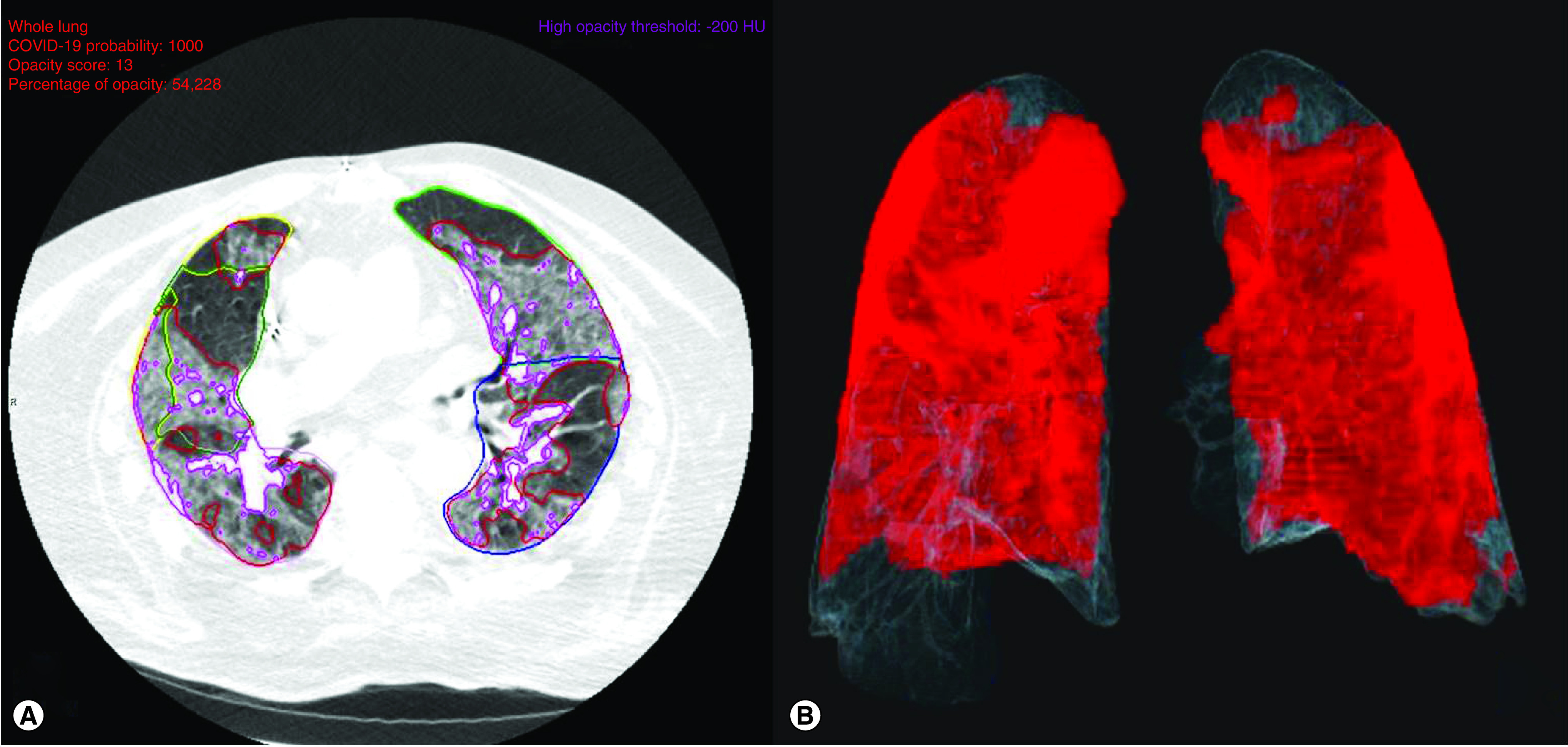
Automated chest computed tomography on admission of an 88-year-old male patient from group III who died during hospitalization. Lung segmentation limits are represented by green, yellow and blue lines. Low (red lines) and high (purple lines) density lesions are also highlighted **(A).** Total opacification percentage and lung volume were calculated at 54.2% and 2.9 l, respectively. **(B)** represents a 3D reconstruction of both lungs, with total opacities shown in red **(B)**.

During the study period, CT examinations were performed on admission using a 64-slice spiral scanner (LightSpeed VCT, GE Healthcare, WI, USA). The protocol was as follows: a tube voltage of 120 kV; smart mA tube current modulation (100–550); pitch of 1.375; reconstruction matrix of 512 × 512; slice thickness of 1.25 mm; and breath hold at full inspiration. Subsequently, tomographic images were recovered and processed by CT Pneumonia Analysis 2.0 and Syngo.via software (VB40 version, Siemens Healthineers, Germany), which automatically identifies and quantifies image patterns for research purposes in COVID-19 and other pulmonary infections, as reported by previous publications [6,7]. Utilizing a noncontrast chest CT image, the software segments the lungs and recognizes opacification patterns before providing 2D and 3D image demonstrations of pulmonary disease burden. Automated information on bilateral pulmonary volumes and opacification percentages were provided, and opacities were further stratified into consolidations (≥ -200 HU) or GG lesions (< -200 HU). The software was also programmed to effectively identify regions with minor motion artefacts, which were subsequently labeled as normal lung. All processed images were reviewed by two experienced radiologists who were blinded to disease severity and biomarker levels. Final automated data were confirmed by complete interobserver agreement. Figure 1A & B represents a processed CT image of a patient included in the study.

Clinical (demographic variables and co-morbidities), laboratory and tomographic data were collected and compared between the four predefined groups. In order to verify the possibility of selection bias for those with CT scans and biomarker measurement, the cohort of excluded patients was also analyzed regarding clinical characteristics and in-hospital outcomes. Clinical outcomes were defined as length of stay, intensive care unit (ICU) admission rate and all cause in-hospital mortality, and were compared between the four biomarker groups.

Stata^®^ 11.0 software was used for statistical analysis. Categorical variables were analyzed with χ^2^ and Fisher’s exact test. All continuous variables were non-normally distributed according to the Shapiro-Wilk test, and were expressed by the median and 25th to 75th percentile interquartile range (IQR). Such data were evaluated by the Wilcoxon–Mann–Whitney and Kruskal–Wallis tests. Graphical models to evaluate associations between quantitative CT data and mortality were represented by predicted probability plots. Variables with significance in the univariate analysis were included in a multivariate logistic regression model to determine independent predictors of death. A p-value <0.05 was considered significant. The study conforms to the guidelines of the Declaration of Helsinki and obtained appropriate institutional review board approval.

## Results

During the study period, 230 hospitalized patients with COVID-19 were screened for inclusion, of which 148 were submitted to chest CT and complete biomarker workup on admission. When compared with those who were excluded from the analysis, there were no significant differences in age, sex or clinical risk factors such as hypertension, diabetes and cardiovascular disease between both cohorts, and outcomes were also similar (Supplementary Table 1). Patients were predominantly males with a median age of 69 years (IQR: 54–82) and an elevated prevalence of hypertension and diabetes, despite a low frequency of known cardiovascular disease. Underlying pulmonary disease and current or previous tobacco use were also uncommon. Median symptom duration before admission was 7 days (IQR: 3–9) and overall oxygen saturation on room air was 93% (IQR: 90–95). Complete clinical and laboratory baseline characteristics are listed in Table 1.

**Table 1.** Clinical, laboratory and tomographic baseline characteristics according to biomarker elevation on admission.

Baseline characteristics	Total (n = 148)	Groups (number of elevated biomarkers^†^)				p-value^‡^
		0 (n = 40)	I (n = 49)	II (n = 47)	III (n = 12)	
Age, median (IQR), years	69 (54–82)	58 (50–72)	67 (52–77)	72 (61–86)	87 (85–90)	**<0.001**
Male, n (%)	85 (57.4)	24 (60)	24 (49.0)	30 (63.8)	7 (58.3)	0.51
BMI, median (IQR), kg/m^2^	26.8 (23.9–29.7)	26.2 (24.5–29.4)	26.3 (23.1–29.5)	27.6 (24.5–30.1)	27.8 (26.9–31.8)	0.30
**Medical history, n (%)**						
Hypertension	75 (50.7)	17 (42.5)	23 (46.9)	29 (61.7)	6 (50.0)	0.30
Diabetes	45 (30.4)	11 (27.5)	13 (26.5)	16 (34.0)	5 (41.7)	0.66
CVD	22 (14.9)	3 (7.5)	7 (14.3)	7 (14.9)	5 (41.7)	0.05
Tobacco use^§^	14 (9.5)	2 (5.0)	3 (6.1)	7 (14.9)	2 (16.7)	0.24
Asthma or COPD	16 (10.8)	4 (10.0)	5 (10.2)	5 (10.6)	2 (16.7)	0.89
Oxygen saturation^¶^, median (IQR), %	93 (90–95)	95 (92–96)	94 (91–95)	92 (88–94)	91 (86–95)	**0.003**
Systolic BP, median (IQR), mmHg	130 (120–145)	132 (119–144)	129 (119–142)	138 (119–149)	130 (124–150)	0.72
**Blood tests, median (IQR)**						
Leukocyte count, cells/mm^3^	6,170 (4540–8320)	5305 (3930–6990)	5700 (4620–7860)	7030 (5280–9290)	7970 (5500–10,370)	**0.01**
Lymphocyte count, cells/mm^3^	904 (635–1190)	960 (766–1245)	915 (669–1221)	832 (527–1218)	870 (606–977)	0.27
Hemoglobin, mg/dl	13.3 (11.6–14.6)	14.5 (13.4–15.1)	13.1 (11.6–14.1)	12.8 (11–14)	11.4 (10.5–12.8)	**<0.001**
C-reactive protein, mg/ml	7.4 (4.2–14.6)	3.3 (1.6–4.9)	7.2 (4.8–14)	14.5 (8.2–18.3)	14.7 (10.8–21.0)	**0.004**
D-dimer, ng/ml	810 (470–1443)	442 (341–592)	745 (465–1086)	1339 (862–1965)	2248 (1275–5556)	**<0.001**
hs-Troponin I, pg/ml	11 (11–24)	<12^††^	11 (11–12)	16 (11–55)	68 (45–450)	**<0.001**
Creatinine, mg/dl	0.9 (0.7–1.1)	0.8 (0.7–1.0)	0.8 (0.6–1.0)	1.0 (0.8–1.4)	1.1 (0.7–2.2)	**0.02**
**Pulmonary opacities^#^, median (IQR), %**						
Total	15.8 (7.1–30.6)	7.5 (2.2–14.9)	17.5 (8.4–27.5)	25.1 (13.8–47.1)	37.1 (21.7–53.6)	**<0.001**
Ground glass lesions (<200 HU)	13.5 (5.9–25.6)	5.4 (2.2–11.9)	13.3 (7.9–24.7)	19 (11–31)	25.8 (19.4–46.4)	**<0.001**
Total lung volumes^#^, median (IQR), l	3.5 (2.9–4.7)	4.1 (3.5–4.8)	3.4 (2.9–4.5)	3.5 (2.7–4.9)	3.0 (2.4–3.3)	**0.02**
**Medications, n (%)**						
Antiplatelets	19 (12.8)	4 (10.0)	4 (8.2)	9 (19.2)	2 (16.7)	0.38
Anticoagulants	17 (11.5)	2 (5.0)	4 (8.2)	7 (14.9)	4 (33.3)	0.05
β-blockers	27 (18.2)	5 (12.5)	6 (12.2)	11 (23.4)	5 (41.7)	0.07
ACEi or ARB	45 (30.4)	10 (25.0)	18 (36.7)	15 (31.9)	2 (16.7)	0.49
Statins	41 (27.7)	10 (25.0)	10 (20.4)	17 (36.2)	4 (33.3)	0.35

† High-sensitivity troponin I, D-dimer and C-reactive protein.

‡ p < 0.05 is considered to indicate statistical significance (bold values).

§ Current or previous.

¶ On ambient air.

# Chest computed tomography analysis.

†† Assay detection threshold.

ACEi: Angiotensin enzyme inhibitor; ARB: Angiotensin receptor blocker; BP: Blood pressure; CVD: Cardiovascular disease; COPD: Chronic obstructive pulmonary disease; hs: High-sensitivity; HU: Hounsfield unit; IQR: Interquartile range.

**Figure 2.**
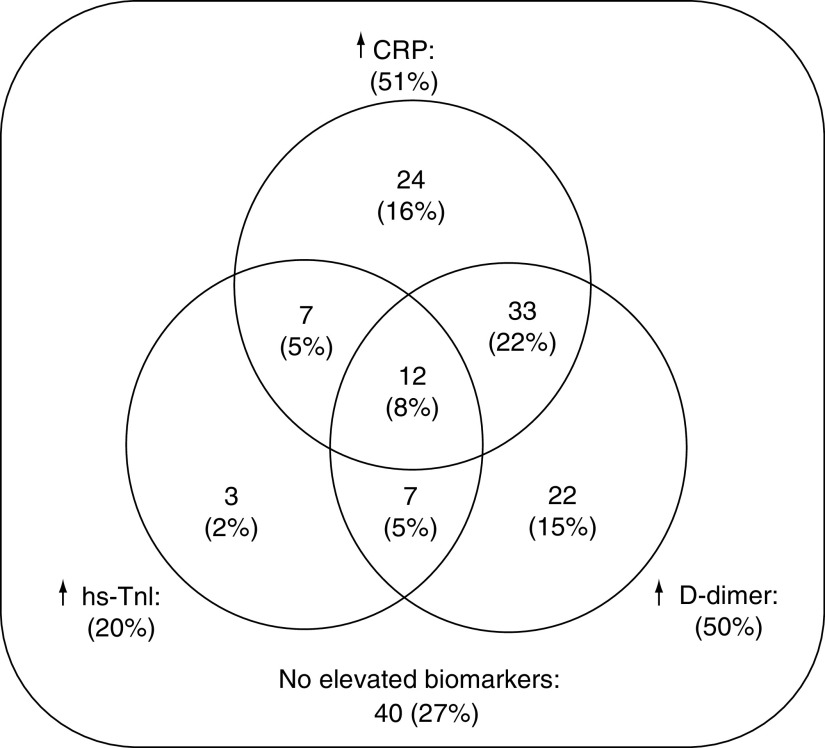
Distribution of patients among the four prespecified groups according to the number of elevated biomarkers on admission (0, I, II or III). CRP: C-reactive protein; hs-TnI: High-sensitivity troponin I.

Among the 148 study patients, 49 (33.1%) had one increased biomarker (group I), 47 (31.8%) presented with two elevations (group II) and 12 (8.1%) individuals had three biomarkers above the prespecified thresholds (group III). The remaining 40 patients (27%) were classified as not having biomarker elevation (group 0). Specific biomarker distribution within each group is presented in Figure 2. Myocardial injury was reported in 20% of cases, in contrast to the expected frequency of elevated CRP (51%) and D-dimer levels (50%) due to the predefined thresholds. As the number of altered biomarkers escalated, median levels within each group also increased.

A significant positive association was observed between median age and the number of altered biomarkers, with a steady progression from group 0 (58 years, IQR: 50–72) to group III (87 years, IQR: 85–90). A decrease in oxygen saturation on admission was also identified as the number of elevated biomarkers increased. Total leukocyte count, hemoglobin and creatinine levels were significantly different among the four groups, with the most altered values recorded in group III (Table 1).

**Figure 3.**
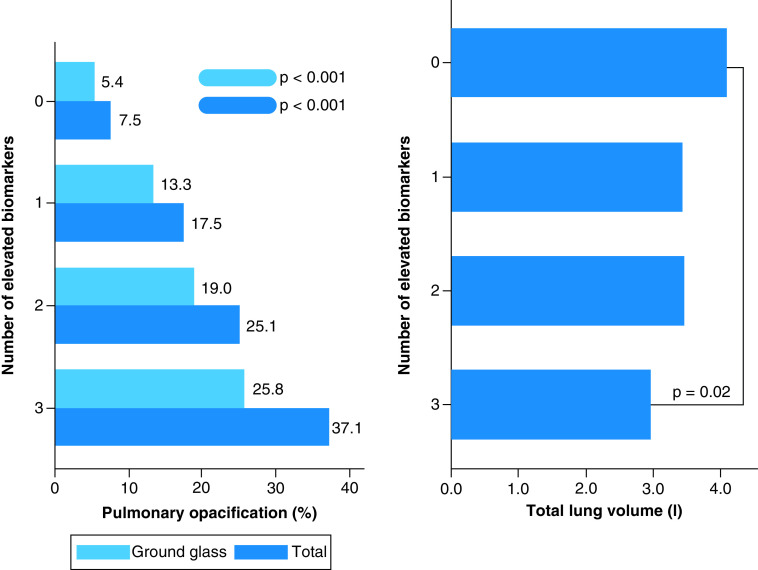
Associations between the number of elevated biomarkers and pulmonary disease burden parameters from automated chest computed tomography on admission (total opacification, ground glass lesions and lung volume).

Although major motion artefacts may affect automated image acquisition and interpretation, none of the included CT scans were compromised by this feature. Median recorded values for total pulmonary opacification, GG lesions and total lung volumes were 15.8% (IQR: 7.1–30.6), 13.5% (IQR: 5.9–25.6) and 3.5 L (IQR: 2.9–4.7), respectively. A positive association was observed between biomarker elevation and pulmonary disease burden. As the number of altered biomarkers increased, a similar tendency was identified regarding total opacification and GG percentages. An inverse trend was recognized concerning total lung volumes, especially when groups 0 and III were compared (Table 1 & Figure 3).

During the hospitalization period, the use of high-flow nasal cannula and noninvasive positive pressure ventilation was similar among those with biomarker elevation, and significantly lower in group 0. A total of 33 (22.3%) patients required mechanical ventilation and this percentage was higher in group III, as was the employment of systemic steroids. Similarly to previous studies, those with total opacification more than 40% were at higher risk for invasive ventilation during hospitalization (OR: 2.69; 95% CI: 1.11–6.51; p = 0.03) [5,6]. Broad spectrum antibiotics, enoxaparin and tocilizumab prescription were not different between the four groups (Supplementary Table 2).

**Figure 4.**
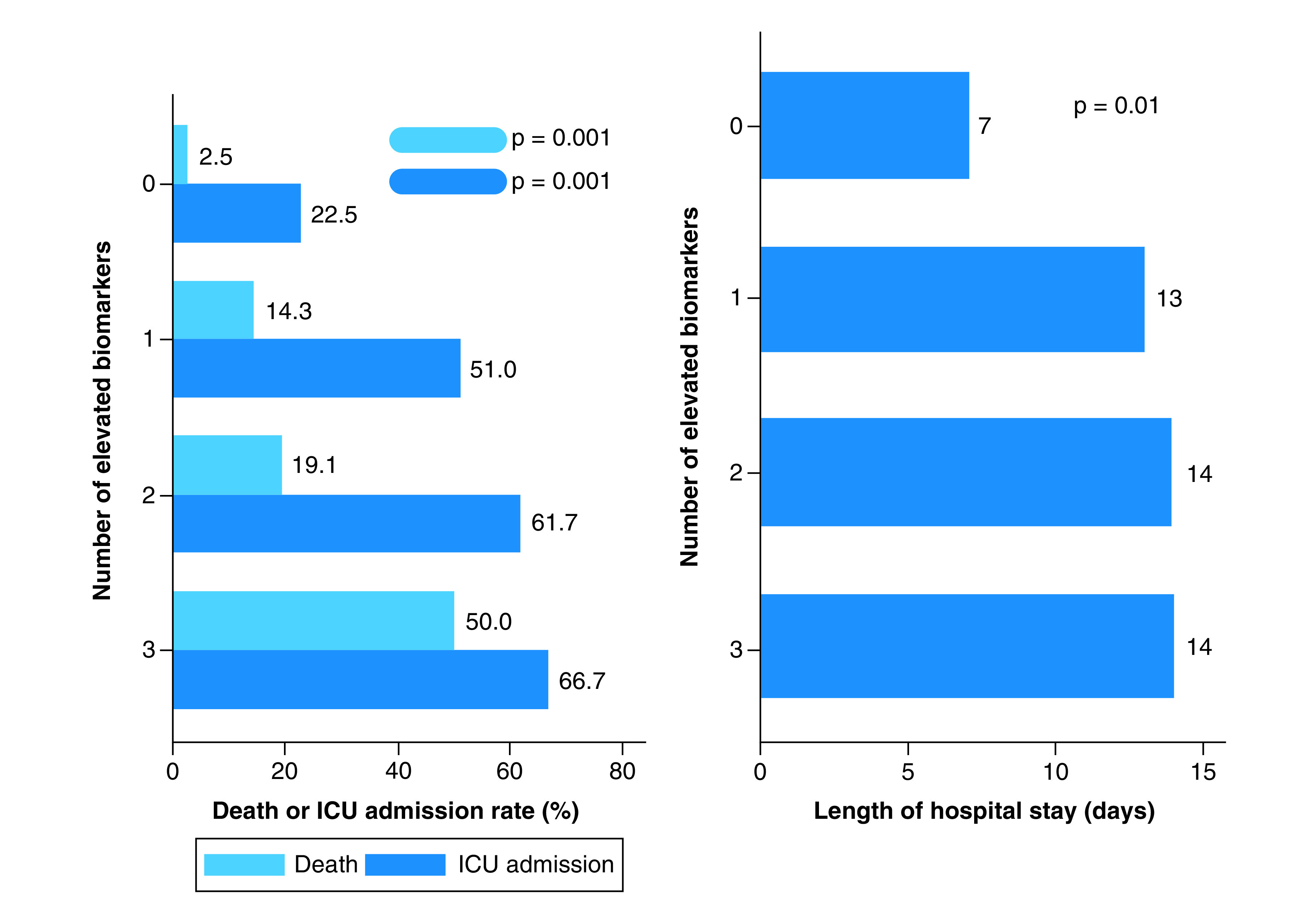
Associations between the number of elevated biomarkers on admission and adverse in-hospital outcomes (death, intensive care unit admission and length of stay). ICU: Intensive care unit.

23 patients (15.5%) died during the hospitalization period. Median length of stay was 12.5 days (IQR: 6–22.5) and 71 patients (48.0%) required ICU admission. In-hospital death and ICU admission rates were significantly associated with the number of elevated biomarkers. The frequency of both outcomes steadily progressed from group 0, reaching a 50% mortality rate in group III. Even among those less than 60 years of age, biomarker elevation was associated with subsequent ICU admission (0: 4.8% vs I: 33.3% vs II: 36.4%; p = 0.046). Length of hospital stay was similar among patients with increased biomarkers, but significantly lower in group 0 (Figure 4).

**Figure 5:**
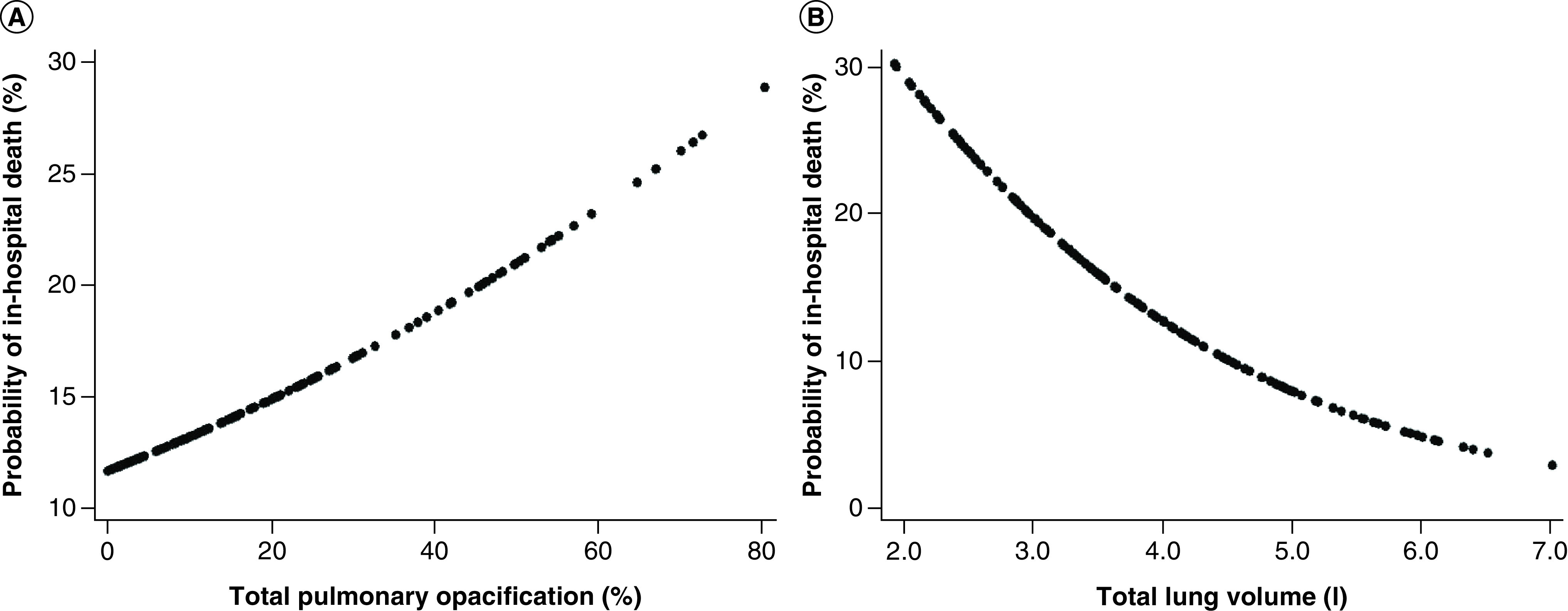
Computed tomography data and mortality. Estimated probability of in-hospital death according to total lung opacification **(A)** and total lung volume **(B)**, obtained from automated chest computed tomography on admission.

**Figure 6.**
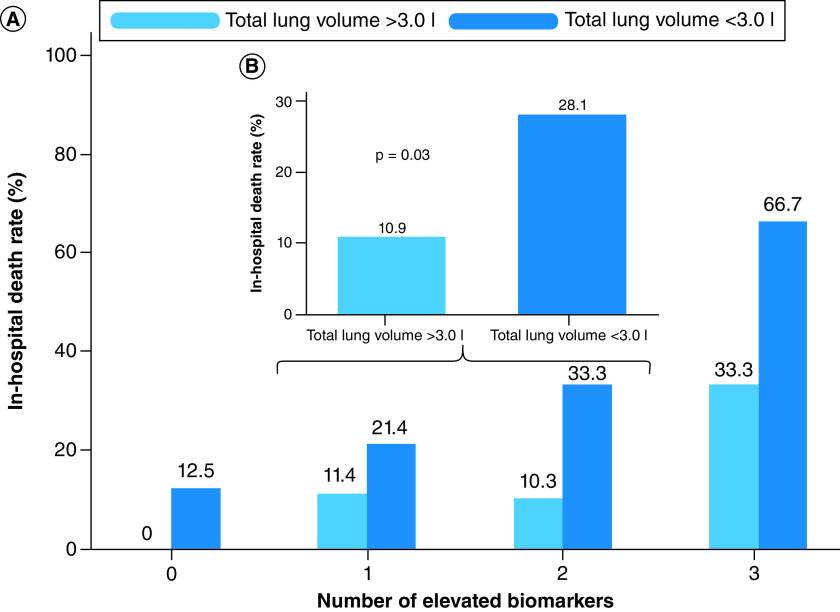
Additional prognostic value of total lung volume and biomarker evaluation. Mortality rates within each biomarker group according to total lung volume quantification **(A)**. Combined analysis of intermediate risk patients (one or two elevated biomarkers, n = 96), demonstrating that lower lung volumes (<3.0 l) significantly discriminated between those with higher and lower risk of death **(B)**.

A positive trend was observed between total pulmonary opacification percentages and death, whereas an inverse relationship with the same outcome was identified regarding median total lung volume (Figure 5A & B), which was significantly lower among nonsurvivors (2.9 l; IQR: 2.4–3.6 vs 3.8 l; IQR: 3.0–4.8; p = 0.006). Automated lung volume quantification provided additional risk stratification for all four biomarker groups, when a cut-off value of 3.0 l was considered. Within each group, those with lower volumes had higher death rates than previously observed. The highest mortality (66.7%) was registered in group III among those with total lung volume less than 3.0 l. Conversely, no deaths occurred when normal biomarker levels and lung volumes were present simultaneously (Figure 6A). The additional prognostic value provided by lung volume quantification was most evident in groups I and II, particularly when they were evaluated in combination. Patients with lower volumes once again had significantly higher death rates (28.1%), even when compared with the 19.1% total mortality recorded in group II (Figure 6B).

In the univariate analysis, predictors of mortality included age, cardiovascular disease, systolic blood pressure (BP), lymphocyte count, hemoglobin level, serum creatinine and total lung volume on CT. Nonsurvivors also expressed higher median levels of hs-TnI (35 pg/ml; IQR: 11–82 vs 11 pg/ml; IQR: 11–15; p < 0.001) and CRP (15 mg/ml; IQR: 7.0–17.9 vs 7.1 mg/ml; IQR: 3.3–14.0; p = 0.003). Although isolated D-dimer levels were not associated with death among all study patients, a borderline result was found among the 83 patients who were prescribed a maximum daily enoxaparin dose ≤40 mg (OR: 1.0; 95% CI: 1.0–1.1; p = 0.016). As such, D-dimer was also included in the multivariate model. In this analysis, only age (OR: 1.14; 95% CI: 1.05–1.24; p = 0.02), systolic BP (OR: 0.96; 95% CI: 0.93–0.99; p = 0.03) and CRP levels (OR: 1.13; 95% CI: 1.02–1.26; p = 0.02), remained as significant predictors of mortality (Table 2).

**Table 2.** Predictors of in-hospital death on univariate and multivariate analyses.

Variable	Univariate analysis OR: (95% CI)	p-value^†^	Multivariate analysis OR (95% CI)	p-value^†^^,^^‡^
Age (years)	1.11 (1.06–1.17)	<0.001	1.14 (1.05–1.24)	**0.002**
Cardiovascular disease	3.21 (1.13–9.07)	0.03	1.14 (0.24–5.43)	0.87
Systolic BP	0.97 (0.95–0.99)	0.04	0.96 (0.93–0.99)	**0.03**
Lymphocyte count	0.99 (0.99–1.0)	0.01	0.99 (0.99–1.0)	0.09
Hemoglobin level	0.78 (0.64–0.94)	0.01	1.09 (0.79–1.5)	0.57
C-reactive protein	1.07 (1.01–1.13)	0.01	1.13 (1.02–1.26)	**0.02**
D-dimer	1.0 (0.99–1.0)	0.13	0.99 (0.99–1.0)	0.23
Elevated hs-Troponin I	6.93 (2.64–18.19)	<0.001	1.03 (0.22–4.74)	0.97
Creatinine	3.35 (1.63–6.87)	0.001	2.27 (0.72–7.16)	0.16
Total lung volume on CT	0.99 (0.99–1.0)	0.02	0.99 (0.99–1.0)	0.54

† p < 0.05 is considered to indicate statistical significance.

‡ Bold values are p < 0.05.

BP: Blood pressure; CT: Computed tomography; hs: High-sensitivity; OR: Odds ratio.

## Discussion

Despite being primarily acknowledged as a viral respiratory disease, COVID-19 has been increasingly recognized as a systemic condition. The disease’s complex inflammatory response inflicts significant ramifications in the immune and coagulation pathways, ultimately promoting widespread endotheliopathy and hypercoagulability [13]. The foremost result is the potential to affect the respiratory system and multiple organs simultaneously, which has a direct impact on clinical outcomes [9–11].

Spiezia *et al.* previously reported significantly elevated fibrinogen and D-dimer levels among 22 patients with COVID-19 and acute respiratory failure, along with hypercoagulable thromboelastometry profiles [12]. Although these findings are a result of COVID-19-associated coagulopathy and may be associated with worse clinical outcomes, additional mechanisms are also involved in clinical deterioration. Systemic inflammation, hypercoagulability and myocardial injury constitute a triad of interrelated pathways, which enhance the deleterious clinical effects promoted by the disease’s respiratory compromise. In this context, CRP, D-dimer and hs-TnI are potential surrogates, which represent an underlying activation of such mechanisms, and thus assist in risk stratification [10]. This approach may improve resource allocation and optimize patient care, which are essential components to successfully manage the current COVID-19 pandemic. Biomarker research is also promising to guide the development of future therapies in this field [13].

The prognostic value of various biomarkers in patients with COVID-19 has been described both individually and using a multiple testing approach [10,11]. C-reactive protein, D-dimer and troponin are thoroughly available and appear to offer complementary information by representing different pathophysiological components of the disease. Previous studies have suggested that TnI elevation is more frequently observed in older individuals with COVID-19, particularly due to the higher frequency of underlying cardiovascular disease. However, this pattern may not be as consistent when D-dimer and CRP elevations are considered solely based on age. Manocha *et al.* published data on 1053 patients with COVID-19 submitted to biomarker evaluation and found that median age was higher in individuals with myocardial injury, but not among those with elevated D-dimer or CRP levels [14]. Nevertheless, older age is an established risk factor for poor outcomes in this context and may be expected to accompany other predictors of adverse events, including biomarker elevation.

Similarly to the current study, Smilowitz *et al.* retrospectively analyzed 2895 consecutive hospitalized patients with COVID-19 with available measurements of all three biomarkers on admission. Myocardial injury was identified in 16.8% of patients, whereas CRP and D-dimer were increased in 78.1 and 77.5%, respectively. A stepwise progression in mortality was also observed according to the number of altered measurements. The three biomarkers were simultaneously elevated in 11.5% of cases, with a 50.9% in-hospital mortality rate attributed to this group. Accordingly, those without any elevation were at low risk of death (3.6%). Although the greater number of patients classified with increased CRP and D-dimer levels could be explained by lower threshold criteria, these results underscore the prognostic value of a simple biomarker strategy performed on hospital admission [10].

In our study, biomarker elevation was also associated with clinical severity. Over 60% of patients with at least two altered results were admitted to the ICU and those in group 0 had the shortest length of hospital stay (7 days; IQR: 3.5–14.5). These findings were also consistent among younger patients (<60 years), suggesting that age is not the only factor that accounts for the prognostic yield of this approach. In addition, as the number of elevated biomarkers increased, median levels for each measurement were also progressively higher. This concurrent progression throughout the groups suggests that although the three biomarkers may represent the involvement of different pathways, they are in fact integrated in a broader systemic response, which ultimately determines the disease’s course. Inflammation, immunothrombosis, endotheliopathy and myocardial injury are inter-related in the complex response triggered by COVID-19 [13].

Despite the advantages and simplicity of the multiple biomarker approach, the extent of respiratory involvement represents an important component of risk stratification, which must be appreciated. Although such evaluation begins on physical examination and determination of oxygen saturation, pulmonary disease burden estimation has been associated with mortality among hospitalized patients in this context. Both quantitative and qualitative CT analyses appear to have prognostic value, and may function as better predictors of outcomes when compared with isolated clinical models. Colombi *et al.* previously reported among 248 patients with COVID-19 that visual pneumonia extent more than 40% on hospital admission was associated with reduced survival [5]. Similarly, a study with 30 hospitalized patients published by Cheng *et al.* also found a positive correlation between quantitative disease burden on CT and both inflammatory parameters and clinical disease severity [3]. Furthermore, quantitative analysis may be superior to semiquantitative scoring for prognostic stratification in this scenario, as reported by Ufuk *et al.* among 76 patients with COVID-19 [15].

Our results provide additional support that quantitative CT parameters may have an important role in predicting hard outcomes. Automated software analysis offered a reliable and consistent means of obtaining relevant prognostic information. Greater pulmonary disease burden was reflected by reduced lung volumes and higher total opacification percentages, which were primarily composed by GG lesions. Previous studies have primarily focused on demonstrating the correlation between automated imaging data and clinical severity scores or surrogate laboratory markers [3,15,16]. In the current study, these parameters were also important predictors of hard outcomes, including mechanical ventilation and in-hospital death, which is essential to potentially guide appropriate treatment decisions.

The degree of pulmonary compromise could possess a twofold significance. While respiratory deterioration is an important component of the pathophysiology, which leads to disease progression, pulmonary lesion severity may also function as a marker of underlying systemic involvement and potential multiorgan complications. Ileri *et al.* recently described the relationship between cardiac biomarkers and CT findings in 140 hospitalized patients with COVID-19 pneumonia. Those with greater pulmonary disease burden presented with higher levels of troponin, although the association was lost when adjusted for age, sex and other inflammatory markers. However, only 53% of the patients were PCR positive and the authors did not evaluate the relationship between enzyme levels, CT findings and prognosis [17].

The stepwise increase in opacification percentages from group 0 to III suggests that respiratory deterioration transpires simultaneously to systemic impairment. Group III also presented lower total lung volumes, which further supports such hypothesis. Although both CT parameters appeared to have prognostic value, reduced lung volume was a more consistent predictor of death. Within all biomarker categories, total lung volume provided further risk stratification considering a threshold <3.0 l, which was associated with higher mortality and underscores the value of adding imaging findings to biomarker evaluation. This analysis was based on previously published data demonstrating a similar cut-off value in COVID-19 patients classified with at least moderate disease [6].

Most importantly, the prognostic impact of reduced lung volume was particularly evident in groups I and II. Though death rates were similar between these groups when values were >3.0 l, mortality increased significantly when volumes were below this threshold (Figure 6A & B). Comparable results have also been previously published by Montenegro *et al.*, reporting that residual lung volume was an independent predictor of death among 138 patients with COVID-19 submitted to automated CT analysis [18]. These outcomes support the concept that automated CT data could provide complementary information to the multiple biomarker approach, especially in clinically stable patients initially classified at low to intermediate risk, such as those in groups I and II. This strategy would contemplate surrogates for inflammation, thrombogenicity, myocardial injury and pulmonary compromise, thus representing a more comprehensive protocol.

When independent associations with death were evaluated in the multivariate model, age, systolic BP and CRP remained as significant predictors. Although this analysis does not underscore the hypotheses created by the previous results, the finding that CRP persisted as a prognostic marker is noteworthy. It is not surprising that in patients who progress to severe clinical disease, a surrogate for systemic inflammation performs as a significant independent predictor of adverse outcomes. Ultimately, the inflammatory pathway possesses ramifications into many other components of the disease’s pathophysiology, including increased thrombogenicity and myocardial injury [13].

This study has limitations, which have to be acknowledged. The retrospective and single-center nature of the results should be confirmed in larger studies. Selection bias cannot be ruled out since 35.6% of patients were not submitted to chest CT and/or full biomarker workup during the study period. However, there were no differences in clinical characteristics or outcomes between the two cohorts, suggesting that the included patients were representative of the total population that was screened. The specific thresholds that were considered for CRP and D-dimer were above the upper reference limit, and may perform differently in other populations with COVID-19. In addition, it is not possible to thoroughly estimate how age may have influenced the results, especially because this variable remained associated with death in the multivariate analysis. Treatment patterns were not standardized, although there were no differences in prescription rates of enoxaparin, tocilizumab or antibiotics between the four groups. Remdesivir was not prescribed in any of the patients since local approval was still pending during the study period. The greater frequency of mechanical ventilation and corticosteroid use in group III, reflects the underlying clinical severity of the disease among those patients. Finally, automated chest CT analysis is not widely available and may not yet represent a practical technology for clinical use. Still, the relevance of the results lie in supporting the concept of an integrated pathophysiology associated with COVID-19, which should be thoroughly evaluated upon hospital admission. As such, future research should focus on providing additional insights into alternative strategies for pulmonary disease burden quantification.

## Conclusion

An initial simple multiple biomarker strategy comprised of CRP, D-dimer and hs-TnI demonstrated promising results for estimating the risk of death and ICU admission in patients hospitalized with COVID-19, although the influence of underlying age differences cannot be excluded. Determination of total pulmonary opacifications and lung volumes from automated quantitative chest CT data provided further prognostic value when added to biomarker evaluation on hospital admission. Future studies are necessary to determine additional associations and optimal thresholds of biomarkers and CT parameters in this context, both for risk stratification and treatment development.

**Summary points**

- In the COVID-19 pandemic, risk stratification is essential to optimize allocation of resources.
- Pulmonary disease burden and biomarkers are possible predictors of outcomes in this scenario and provide complementary clinical information.
- Combining troponin I, D-dimer and C-reactive protein levels with automated quantitative chest computed tomography (CT) data has not been previously evaluated for risk stratification in COVID-19.
- A retrospective cohort of hospitalized COVID-19 patients was studied, analyzing clinical information, biomarker levels, quantitative chest CT data and adverse in-hospital outcomes.
- A total of 230 patients were screened of which 148 had biomarker and imaging data on admission.
- One, two or three elevated biomarkers were found in 33.1, 31.8 and 8.1% of patients, respectively.
- The number of elevated biomarkers was significantly associated with greater opacification percentages, lower lung volumes and higher death rates.
- Total lung volume <3.0 l provided complementary stratification to biomarker evaluation for mortality, especially among patients with one or two altered biomarkers.
- Adding automated quantitative CT data to a multiple biomarker approach could offer further prognostic information and aid in clinical management of COVID-19 patients.
