# Supplementary material for: Biomarker evaluation for prognostic stratification of patients with COVID-19: the added value of quantitative chest CT
Source: Biomark Med. 2022 Feb 18:10.2217/bmm-2021-0536. doi: 10.2217/bmm-2021-0536 (PMC8855956; doi:10.2217/bmm-2021-0536)
Supplement: Supplementary file 1 [file Supplementary_tables.docx]

**Supplementary tables**

| **Patient characteristics and outcomes** | **With biomarkers and CT scan (%) (n=148)** | **Without biomarkers and CT scan (%) (n=82)** | ***p* value^a^** |
| --- | --- | --- | --- |
| Age, median (IQR), y  Male, No. (%)  Hypertension, No. (%)  Diabetes, No. (%)  Cardiovascular disease, No. (%)  Symptom duration, median (IQR), d  MV during hospitalization, No. (%)  In-hospital death, No. (%) | 69 (54-82)  85 (57.4)  75 (50.7)  45 (30.4)  22 (14.9)  7 (3-9)  33 (22.3)  23 (15.5) | 68 (50-81)  47 (57.3)  44 (53.7)  22 (26.8)  12 (14.6)  6 (3-10)  14 (17.1)  14 (17.1) | 0.53  0.99  0.67  0.57  0.96  0.68  0.35  0.76 |

**Table 1.** Clinical characteristics and in-hospital mortality according to biomarker and tomographic workup among 230 patients hospitalized with COVID-19.

^a^*p*<0.05 is considered to indicate statistical significance. CT, computed tomography; IQR, interquartile range; MV, mechanical ventilation

**Table 2.** Pharmacologic and non-pharmacologic treatment patterns during hospitalization according to biomarker elevation on admission.

| **Management during hospitalization** | **Total**  **(n=148)** | **Groups**  **(number of elevated biomarkers^a^)** | | | | ***p***  **value^b^** |
| --- | --- | --- | --- | --- | --- | --- |
|  |  | **0**  **(n=40)** | **I**  **(n=49)** | **II**  **(n=47)** | **III**  **(n=12)** |  |
| NIPPV or HFNC, No (%)  Mechanical ventilation, No. (%)  Systemic corticosteroids, No. (%)  Tocilizumab, No. (%)  Enoxaparin, No. (%)  Daily dose^c^, median (IQR), mg  Antibiotics^d^, No. (%) | 59 (39.9)  33 (22.3)  72 (48.7)  19 (12.8)  142 (96)  40 (40-60)  127 (85.8) | 7 (17.5)  2 (5.0)  12 (30.0)  2 (5.0)  38 (95.0)  40 (40-60)  31 (77.5) | 23 (46.9)  14 (28.6)  27 (55.1)  6 (12.2)  47 (95.9)  60 (40-60)  44 (89.8) | 24 (51.1)  11 (23.4)  22 (46.8)  8 (17.0)  46 (97.9)  50 (40-80)  42 (89.4) | 5 (41.7)  6 (50.0)  11 (91.7)  3 (25.0)  11 (91.7)  60 (20-60)  10 (83.3) | **0.005**  **0.002**  **0.001**  0.17  0.24  0.16  0.32 |

^a^high-sensitivity troponin I, D-dimer and C-reactive protein; ^b^*p*<0.05 is considered to indicate statistical significance (bold values); ^c^highest recorded daily dose; ^d^amoxicillin/clavulanic acid, ceftriaxone, levofloxacin, moxifloxacin, cefepime, piperacillin/tazobactam or meropenem;. HFNC, high-flow nasal cannula; NIPPV, non-invasive positive pressure ventilation.
